# Tibialis anterior surface electromyography during ankle dorsiflexion as a complementary assessment measure after stroke

**DOI:** 10.3389/fneur.2026.1913483

**Published:** 2026-09-10

**Authors:** Jie Zhang, Yujin Zhao, Zhen Tian, Rukun Dou, Chunxiao Wan

**Affiliations:** 1 Tianjin Medical University General Hospital, Tianjin, China; 2 First Teaching Hospital of Tianjin University of Traditional Chinese Medicine, Tianjin, China; 3 National Clinical Research Center for Chinese Medicine, Tianjin, China; 4 Department of Physical and Rehabilitation Medicine, Tianjin Medical University General Hospital, Tianjin, China

**Keywords:** ankle dorsiflexion, average rectified value, lower-limb function, neurorehabilitation, stroke, surface electromyography, tibialis anterior

## Abstract

**Background:**

The Fugl–Meyer Assessment of Lower Extremity (FMA-LE), Berg Balance Scale (BBS), and Brunnstrom lower-limb stage summarize lower-limb motor and balance function after stroke but do not directly reflect the electrical activity of distal muscles during a specific task. The tibialis anterior (TA) is a primary superficial agonist during active ankle dorsiflexion, and its surface electromyography (sEMG) may provide muscle-specific and task-specific complementary information relevant to foot drop and voluntary distal ankle-foot control.

**Objective:**

To examine the relationships of TA sEMG measures obtained during active ankle dorsiflexion with lower-limb motor function, balance, and stage of motor recovery in inpatients with ischemic or hemorrhagic stroke within 1 year of onset, and to explore whether short-term improvements in clinical scales were accompanied by corresponding changes in sEMG measures.

**Methods:**

This single-center observational study included 118 participants with supratentorial or infratentorial index stroke lesions. Bilateral TA and gastrocnemius sEMG was recorded during a standardized seated active ankle dorsiflexion task. The primary measures were paretic-side TA average rectified value (ARV) and the TA paretic/non-paretic ratio. Baseline sEMG analyses included 110 participants, clinical longitudinal analyses included 88, and sEMG longitudinal analyses included 81. Spearman correlation, multivariable linear regression, and paired Wilcoxon signed-rank tests were used, with adjustment for multiple comparisons.

**Results:**

At baseline, paretic-side TA ARV was lower than non-paretic-side TA ARV. Paretic-side TA ARV showed moderate positive correlations with the FMA-LE, BBS, and Brunnstrom lower-limb stage (*ρ* = 0.556, 0.492, and 0.496, respectively), as did the TA paretic/non-paretic ratio (*ρ* = 0.566, 0.440, and 0.417, respectively; all adjusted *q* < 0.001). Both TA measures remained associated with the FMA-LE and BBS after adjustment for age, sex, stroke type, time from stroke onset to baseline assessment, and paretic side. The FMA-LE, BBS, and Brunnstrom lower-limb stage improved during inpatient rehabilitation, whereas no sEMG measure showed a significant longitudinal change after FDR correction.

**Conclusion:**

TA sEMG during active ankle dorsiflexion was associated with concurrent lower-limb motor and balance function and may provide muscle-specific and task-specific information complementary to routine clinical scales. Its longitudinal responsiveness and value in clinical practice require further investigation.

**Clinical trial registration:**

https://itmctr.ccebtcm.org.cn/mgt/search, identifier ITMCTR2026000773.

## 1 Introduction

Lower-limb motor and balance impairments after stroke commonly reduce standing stability, complicate transfers, decrease walking efficiency, and increase safety risks, making them important targets of assessment and treatment in inpatient neurorehabilitation (1–3). These impairments may involve weakness, altered muscle tone, limited selective motor control, abnormal muscle synergies, impaired postural and balance control, and altered coordination and compensation across the hip, knee, and ankle (2, 3). Impaired ankle dorsiflexion is a clinically meaningful distal manifestation that may present as foot drop and compromise toe clearance during swing, foot control at initial contact, and walking safety (4). Paretic ankle dorsiflexor strength is associated with walking speed in independently ambulatory people after stroke, indicating that dorsiflexion is relevant not only to local joint movement but also to overall mobility (5). The TA is one of the primary superficial muscles involved in active ankle dorsiflexion. After stroke, the TA may exhibit impaired motor-unit rate coding, abnormal firing behavior during fatiguing contractions, and altered spinal motoneuron excitability related to ankle-foot motor control (6–8). Thus, the TA is not the only target of lower-limb impairment after stroke, but it provides an anatomically and functionally specific neuromuscular window into voluntary distal ankle-foot control.

The FMA-LE, BBS, and Brunnstrom lower-limb stage are commonly used and clinically meaningful assessments of lower-limb motor and balance function after stroke (3, 9–12). These measures summarize lower-limb motor impairment, balance performance, and stage of motor recovery from different perspectives and support clinical communication and follow-up. However, their total scores or stages cannot determine whether a functional limitation primarily reflects proximal stability, distal selective motor control, abnormal synergy, or altered recruitment of a specific muscle during a defined task. They also do not directly quantify TA sEMG amplitude or bilateral asymmetry during active ankle dorsiflexion. Improvement in a clinical scale may reflect motor recovery, better task performance, or compensatory strategies. sEMG non-invasively records muscle electrical activity during movement and has been used to study motor control and functional assessment in neurorehabilitation (13–18). During standardized active ankle dorsiflexion, paretic-side TA ARV describes task-evoked TA sEMG amplitude, whereas the TA paretic/non-paretic ratio describes within-participant bilateral asymmetry in TA amplitude. TA sEMG therefore provides muscle-specific and task-specific information that should complement, rather than replace, clinical scales.

Previous studies of ankle dorsiflexion biomechanics, post-stroke ankle-foot neuromuscular status, and sEMG-based ankle movement decoding indicate that ankle-foot function is closely related to lower-limb functional performance after stroke (4, 19, 20). However, evidence remains limited on the relationships of TA sEMG measures obtained during standardized active ankle dorsiflexion with the FMA-LE, BBS, and Brunnstrom lower-limb stage in patients undergoing inpatient rehabilitation. It is also unclear whether clinical scales and TA sEMG show similar patterns of short-term longitudinal change during inpatient rehabilitation. We therefore examined the relationships of paretic-side TA ARV and the TA paretic/non-paretic ratio with lower-limb motor function, balance, and stage of motor recovery, and explored whether short-term improvements in clinical scales were accompanied by corresponding changes in the primary TA sEMG measures. We hypothesized that higher baseline task-evoked paretic-side TA sEMG amplitude and a higher TA paretic/non-paretic ratio would be associated with better FMA-LE, BBS, and Brunnstrom lower-limb stage. Gastrocnemius activity and TA–gastrocnemius coactivation measures were treated as secondary or exploratory analyses.

## 2 Materials and methods

### 2.1 Study design and setting

This single-center observational study was conducted in the Department of Rehabilitation at the First Teaching Hospital of Tianjin University of Traditional Chinese Medicine. The analysis had two components. First, admission baseline data were used in cross-sectional analyses of the relationships between TA sEMG measures and concurrent lower-limb motor and balance function. Second, paired admission and discharge data from the same inpatient stay were used in exploratory short-term longitudinal analyses to describe whether changes in clinical scales were accompanied by changes in sEMG measures. The study was approved by the Ethics Committee of the First Teaching Hospital of Tianjin University of Traditional Chinese Medicine (TYLL2026[Z]016). All participants or their legal representatives provided written informed consent. Participants were enrolled between February 10 and June 10, 2026. The study followed the Declaration of Helsinki and was reported in accordance with the STROBE statement (21). This observational analysis was conducted within a prospectively registered parent study (International Traditional Medicine Clinical Trial Registry, ITMCTR2026000773).

### 2.2 Participants

Inpatients with stroke admitted to the rehabilitation department were screened consecutively. Eligibility criteria were a clinical and imaging diagnosis of ischemic or hemorrhagic stroke, no more than 1 year from the index stroke to baseline assessment, and the ability to understand and cooperate with clinical functional assessments and sEMG acquisition.

Patients were excluded if severe cognitive impairment, aphasia, or impaired consciousness prevented them from understanding or performing the test instructions; if a musculoskeletal disorder, peripheral neuropathy, or myopathy materially affected lower-limb motor function; if bilateral paresis or another neurological disorder affected lower-limb motor control; or if lower-limb skin damage, severe edema, or another condition interfered with electrode attachment or signal acquisition. Each analysis set was formed according to the actual availability of the relevant variables; participants were not selected according to discharge outcomes or treatment response.

### 2.3 Clinical and demographic data

Age, sex, stroke type, index lesion location, paretic side, time from stroke onset to baseline assessment, and comorbidities were recorded. Index lesion location was determined from imaging information available at enrollment and classified as supratentorial or infratentorial; infratentorial lesions included brainstem and cerebellar lesions. Baseline clinical scales and sEMG assessments were completed at admission, and the discharge reassessment was uniformly completed 14 days after admission to inpatient rehabilitation. Cross-sectional analyses used baseline data, whereas longitudinal analyses compared baseline with the 14-day assessment from the same inpatient stay.

### 2.4 Standardized active ankle dorsiflexion task

Participants were seated with the hips and knees flexed to approximately 90°, both feet placed naturally, and the ankles maintained as close as possible to neutral. The paretic and non-paretic sides were defined according to the clinical side of paresis and were tested separately. In response to a standardized verbal instruction, participants performed active ankle dorsiflexion to their maximum available range. Five dorsiflexion trials were completed on each side; each contraction lasted 3 s and was followed by 3 s of relaxation. Ipsilateral TA and gastrocnemius sEMG was recorded simultaneously during each trial.

### 2.5 sEMG acquisition and processing

Bilateral TA and gastrocnemius sEMG was recorded using a MyoMove-EOW surface electromyography system (Shanghai NCC Electronic Co., Ltd., Shanghai, China). Before acquisition, the skin over the electrode sites was cleaned and local hair was removed when necessary to reduce skin impedance and motion artifacts. Electrode placement followed SENIAM recommendations (22). Bipolar surface electrodes were aligned with the muscle fibers with an interelectrode distance of approximately 20 mm. TA electrodes were positioned near the proximal one-third of the line between the fibular head and medial malleolus. Gastrocnemius signals were recorded from the medial head, with electrodes placed over the most prominent region of the medial gastrocnemius belly along the longitudinal axis of the calf. The reference electrode was placed over a bony landmark away from the recorded muscles.

sEMG was sampled at 2,048 Hz. After acquisition, recordings were processed using the standardized preset workflow of the device software. According to technical information provided by the manufacturer, the workflow included 10–500 Hz band-pass filtering, 50 Hz notch filtering, full-wave rectification, and smoothing/envelope extraction. The software-derived ARV and integrated electromyography (iEMG) represented the mean absolute amplitude and time integral, respectively, of the rectified signal within the software-defined analysis window. Values from the five active ankle dorsiflexion trials were averaged for analysis. Original EDF files were retained for waveform review and data traceability. Recordings without a valid software export or with signal quality insufficient for metric calculation were treated as missing in the corresponding analysis.

Maximum voluntary contraction (MVC) normalization was not performed. Some inpatients with stroke could not complete a stable and reliable MVC test, and the study was not designed to compare absolute maximal activation capacity across individuals. ARV was therefore interpreted only as task-specific sEMG amplitude during standardized active ankle dorsiflexion and not as a direct measure of muscle strength, maximal neuromuscular activation, or the degree of neurological recovery.

**Figure 1:**
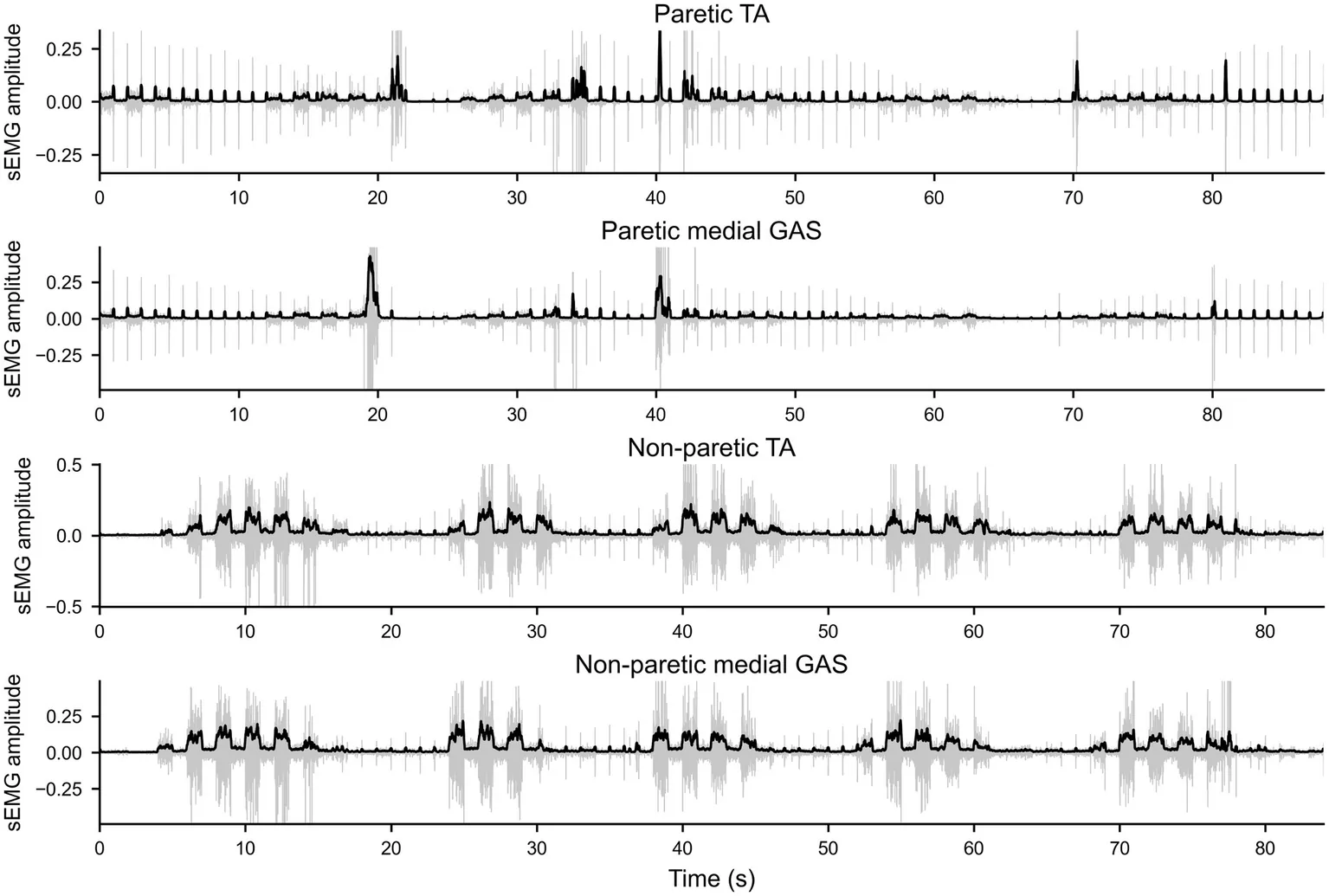
Representative baseline sEMG waveform during the standardized active ankle dorsiflexion task. The representative recording was selected to illustrate typical task-related waveform patterns. Paretic and non-paretic limbs were recorded in separate task trials. Gray traces show processed sEMG waveforms, and black traces show the smoothed envelope. The waveform is shown only as an illustrative example and was not used to calculate the statistical results. GAS, gastrocnemius; sEMG, surface electromyography; TA, tibialis anterior.

Figure 1 provides an example of a baseline sEMG recording obtained during the standardized active ankle dorsiflexion task.

### 2.6 sEMG measures

The primary sEMG measures were paretic-side TA ARV and the TA paretic/non-paretic ratio. The former describes task-specific paretic TA amplitude during active ankle dorsiflexion, whereas the latter describes within-participant asymmetry in bilateral TA amplitude.

$$\begin{array}{l} \mathrm{TA}\;\text{paretic}/\text{non}-\text{paretic ratio}\\ =\text{paretic}-\text{side}\;\mathrm{TA}\;\mathrm{ARV}/\text{non}-\text{paretic}-\text{side}\;\mathrm{TA}\;\mathrm{ARV} \end{array}$$

Secondary sEMG measures included paretic-side gastrocnemius ARV and the gastrocnemius paretic/non-paretic ratio. Exploratory coactivation measures included the co-contraction index (CCI) and coactivation index 1 (CoA1), which were used to describe relative co-recruitment of the TA and gastrocnemius with reference to previous studies (16, 23–25).

CCI=[2×min(ARVTA,ARVGAS)]/(ARVTA+ARVGAS)

CoA1=[2×ARVGAS/(ARVTA+ARVGAS)]×100

ΔsideCoA1=paretic−sideCoA1−non−paretic−sideCoA1

### 2.7 Clinical outcome assessments

Clinical assessments included Brunnstrom lower-limb stage, the FMA-LE, and the BBS. Brunnstrom staging was used to describe the stage of motor recovery of the paretic lower limb (10), the FMA-LE to assess lower-limb motor function after stroke (11), and the BBS to assess balance (12). All three were routine clinical assessments and were interpreted together with sEMG measures to characterize current functional status.

### 2.8 Statistical analysis

Eligible inpatients with analyzable data were enrolled consecutively during the predefined study period; no target sample size was prespecified for a single hypothesis test. Continuous variables are presented as medians and interquartile ranges, and categorical variables as counts and percentages. Baseline paretic versus non-paretic comparisons and longitudinal admission versus discharge comparisons used paired Wilcoxon signed-rank tests with effect size *r*.

Relationships between sEMG measures and clinical scales were assessed using Spearman rank correlations, with 95% confidence intervals estimated using 5,000 percentile bootstrap resamples. Correlations were interpreted as weak, moderate, or strong for |*ρ*| values of 0.10–0.39, 0.40–0.69, and ≥0.70, respectively. Multivariable linear regression was used to assess adjusted associations of the primary TA measures with the FMA-LE and BBS. Models included age, sex, stroke type, time from stroke onset to baseline assessment, and paretic side, and used HC3 robust standard errors.

Multiple comparisons were adjusted within prespecified test families using the Holm method or the Benjamini–Hochberg false discovery rate procedure; the exact test family is defined in the corresponding table note. Analyses of CoA1/CCI, correlations between change scores, and stratified and interaction analyses by stroke type, index lesion location, time since stroke, and baseline severity were exploratory. Missing data were handled using available-case analysis. All tests were two-sided, with *p* < 0.05 considered nominally significant; interpretation prioritized adjusted *p* values or *q* values.

Fixed-sample-size sensitivity analyses estimated the minimum detectable effect at a two-sided *α* of 0.05 and 80% power. All longitudinal sEMG analyses were considered exploratory. Statistical analyses were performed using Python 3.14.4, primarily with NumPy 2.4.4, SciPy 1.17.1, and statsmodels 0.14.6.

## 3 Results

### 3.1 Participants and analysis sets

A total of 118 participants with stroke were included: 83 men (70.3%) and 35 women (29.7%), with a median age of 63.00 [56.25, 70.00] years. Ninety-six participants (81.4%) had ischemic stroke and 22 (18.6%) had hemorrhagic stroke. The index lesion was supratentorial in 84 participants (71.2%) and infratentorial in 34 (28.8%). The paretic side was left in 68 participants (57.6%) and right in 50 (42.4%). The median time from stroke onset to baseline assessment was 26.50 [12.0, 60.8] days (Table 1).

**Table 1:** Demographic and clinical characteristics of the study cohort.

Characteristic	Overall cohort (*N* = 118)
Age, years	63.00 [56.25, 70.00]
Sex	
Male	83 (70.3%)
Female	35 (29.7%)
Stroke type	
Ischemic	96 (81.4%)
Hemorrhagic	22 (18.6%)
Index lesion location	
Supratentorial lesion	84 (71.2%)
Infratentorial lesion	34 (28.8%)
Paretic side	
Left	68 (57.6%)
Right	50 (42.4%)
Time from stroke onset to baseline assessment, days	26.5 [12.0, 60.8]
Baseline FMA-LE score	23.0 [20.0, 27.0]
Baseline BBS score	16.5 [6.0, 31.0]
Baseline Brunnstrom stage	4.0 [3.0, 4.0]
Hypertension	91 (77.1%)
Diabetes mellitus	52 (44.1%)

Values are median [IQR] or *n* (%). The overall cohort included 118 participants. Baseline clinical scores describe the overall cohort at enrollment. FMA-LE, Fugl-Meyer Assessment of Lower Extremity; BBS, Berg Balance Scale; IQR, interquartile range.

**Figure 2:**
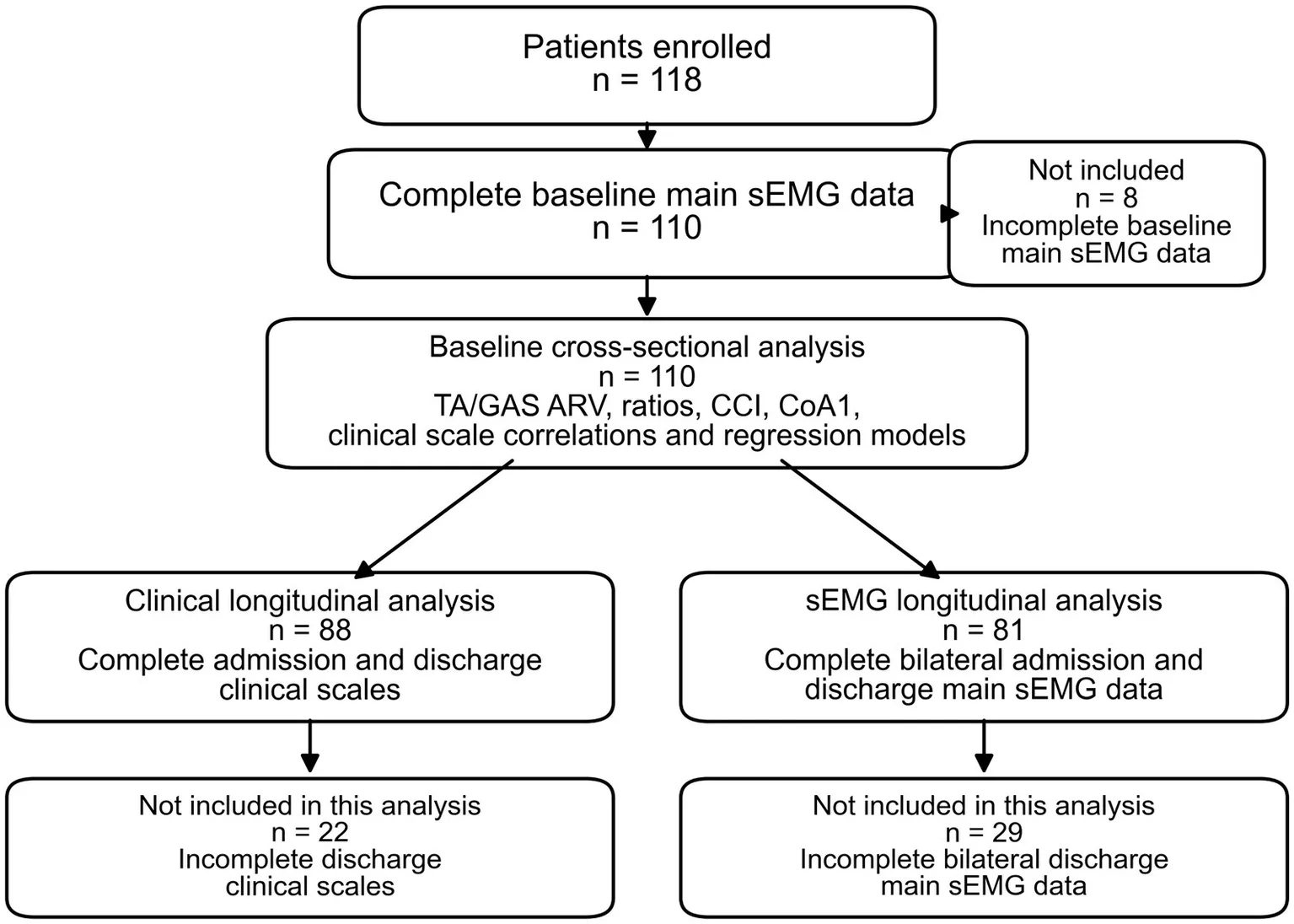
Participant flow and analysis sets. The overall cohort included 118 participants. Baseline sEMG analyses included 110 participants with complete bilateral baseline TA and GAS ARV data. Clinical longitudinal analyses included 88 participants with paired clinical assessments, and sEMG longitudinal analyses included 81 participants with complete bilateral baseline and discharge sEMG data.

Baseline cross-sectional analyses included 110 participants with complete bilateral baseline sEMG data. Clinical longitudinal analyses included 88 participants with complete admission and discharge clinical assessments, and sEMG longitudinal analyses included 81 participants with complete bilateral admission and discharge sEMG data (Figure 2). Participants with and without longitudinal clinical data differed in paretic-side distribution (*p* = 0.003), whereas the remaining prespecified baseline comparisons were not statistically significant. None of the prespecified baseline comparisons between participants with and without longitudinal sEMG data reached *p* < 0.05 (Supplementary Table S1).

### 3.2 Baseline paretic versus non-paretic sEMG comparisons

**Figure 3:**
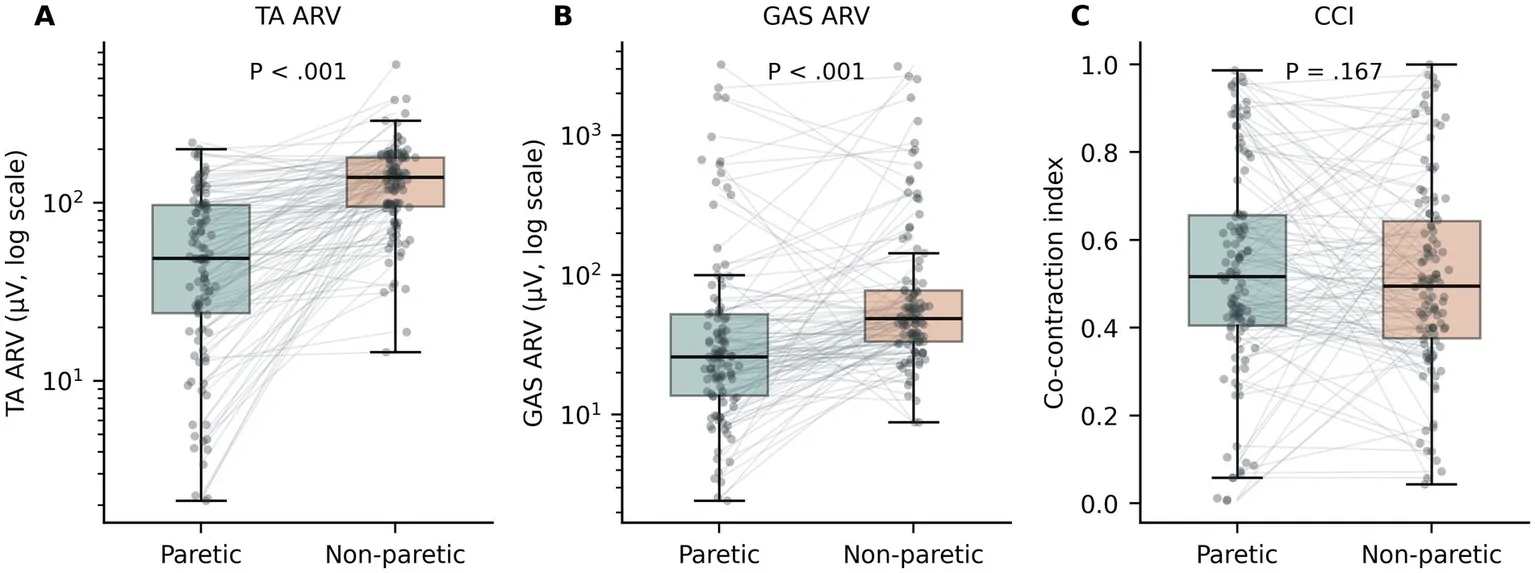
Panels **A–C** show TA ARV, GAS ARV and CCI, respectively. Baseline paretic- and non-paretic-side sEMG comparisons. TA ARV and GAS ARV are displayed on logarithmic *y*-axes to improve visualization of skewed distributions; statistical tests were performed on the original values. CCI is displayed on a linear *y*-axis. Lines connect paired observations from the same participant.

In the baseline sEMG cohort (*n* = 110), paretic-side TA ARV was lower than non-paretic-side TA ARV [48.69 (23.98, 97.13) vs. 139.26 (95.25, 179.24) μV; Holm-adjusted *p* < 0.001; Table 2; Figure 3]. Paretic-side gastrocnemius ARV was also lower than the non-paretic value [25.89 (13.72, 52.55) vs. 48.86 (33.29, 77.54) μV; Holm-adjusted *p* < 0.001; Table 2; Figure 3]. The between-side difference in CCI was not statistically significant (Holm-adjusted *p* = 0.167; Figure 3), whereas paretic-side CoA1 was higher than non-paretic-side CoA1 [61.73 (45.18, 109.85)% vs. 55.04 (43.00, 87.49)%; Holm-adjusted *p* = 0.006; Table 2]. Complete between-side comparisons are provided in Supplementary Table S2.

**Table 2:** Baseline paretic−non-paretic side differences in sEMG measures (*n* = 110).

sEMG measure	Paretic side, median [IQR]	Non-paretic side, median [IQR]	Paretic−non-paretic difference, median [IQR]	Wilcoxon *Z*	Holm-adjusted *p*	*r*
TA ARV, μV	48.69 [23.98, 97.13]	139.26 [95.25, 179.24]	−59.75 [−122.68, −24.49]	−8.441	<0.001	0.805
GAS ARV, μV	25.89 [13.72, 52.55]	48.86 [33.29, 77.54]	−21.24 [−44.95, −2.36]	−5.092	<0.001	0.486
CCI, unitless	0.52 [0.41, 0.66]	0.50 [0.38, 0.64]	0.02 [−0.08, 0.14]	1.382	0.167	0.132
CoA1, %	61.73 [45.18, 109.85]	55.04 [43.00, 87.49]	6.25 [−6.95, 34.02]	2.969	0.006	0.283

Values are medians [IQR]. Paretic−non-paretic difference was defined as paretic side minus non-paretic side. Two-sided Wilcoxon signed-rank tests were used; *p* values were Holm-adjusted across the four measures. Effect size *r* was calculated as |Z|/sqrt(*n*). sEMG, surface electromyography; TA, tibialis anterior; GAS, gastrocnemius; ARV, average rectified value; CCI, co-contraction index; CoA1, coactivation index 1; IQR, interquartile range.

### 3.3 Associations of primary TA measures with clinical scales

**Figure 4:**
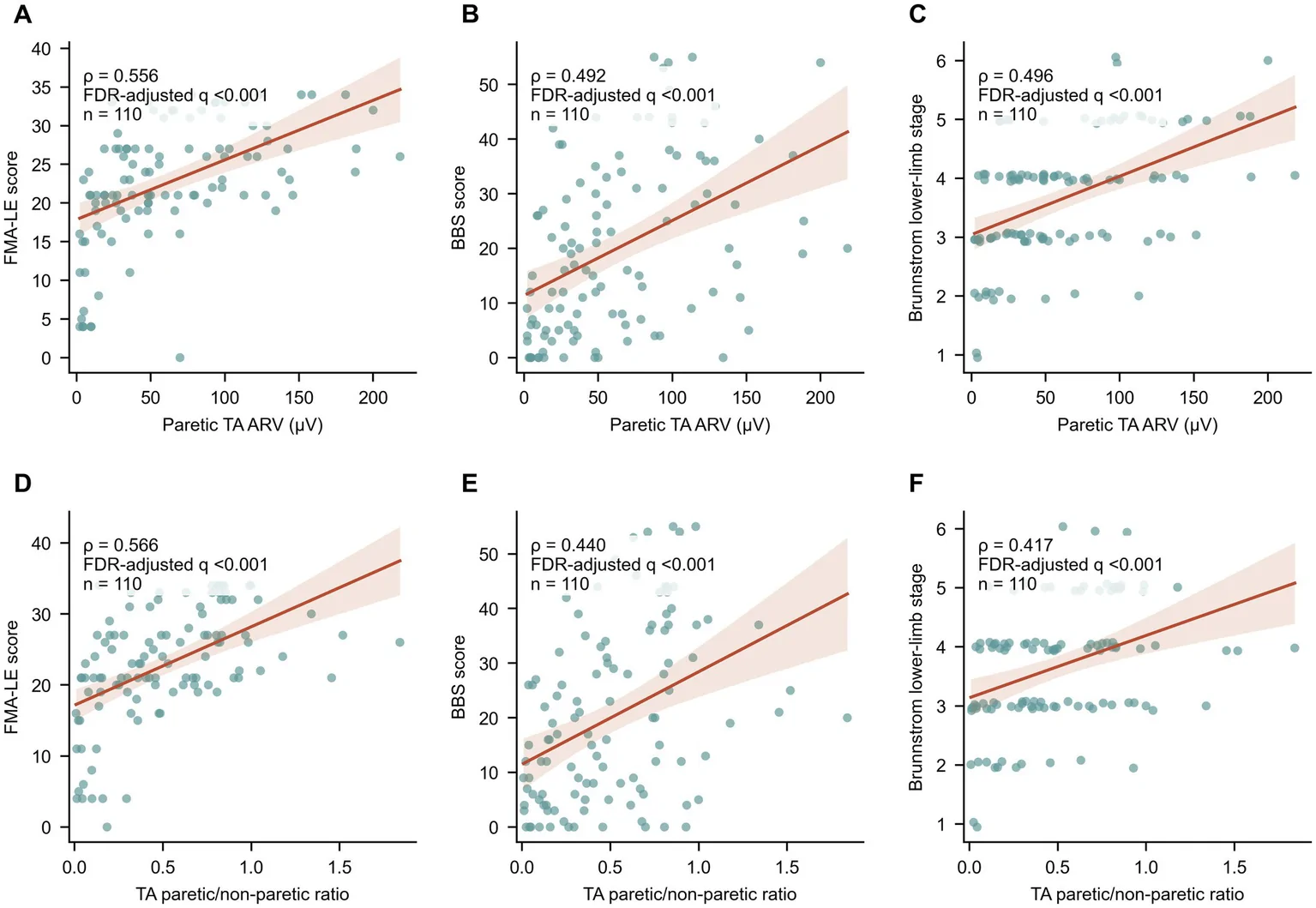
Associations of TA activation measures with clinical function at baseline. Panels **(A–C)** show paretic-side TA ARV versus FMA-LE, BBS, and Brunnstrom stage; panels **(D–F)** show the TA paretic/non-paretic ratio versus the same clinical measures. Solid lines represent ordinary least-squares fits, and shaded areas indicate 95% confidence intervals for the fitted mean; these elements are shown for visualization only. Inferential statistics were based on Spearman rank correlations. *q* values were obtained using the Benjamini–Hochberg false discovery rate correction across the complete 36-test baseline sEMG–clinical correlation family. Brunnstrom values were jittered to reduce overlap.

Both primary TA measures showed moderate positive correlations with lower-limb motor function, balance, and stage of motor recovery. Paretic-side TA ARV correlated with the FMA-LE (*ρ* = 0.556, 95% CI 0.399–0.682), BBS (*ρ* = 0.492, 95% CI 0.328–0.625), and Brunnstrom lower-limb stage (*ρ* = 0.496, 95% CI 0.326–0.636). The TA paretic/non-paretic ratio correlated with the FMA-LE (*ρ* = 0.566, 95% CI 0.422–0.682), BBS (*ρ* = 0.440, 95% CI 0.275–0.583), and Brunnstrom lower-limb stage (*ρ* = 0.417, 95% CI 0.262–0.564). All six BH-FDR-adjusted *q* values were <0.001 (Table 3; Figure 4). Complete cross-sectional correlations between sEMG measures and clinical scales are provided in Supplementary Table S3.

**Table 3:** Associations of TA and coactivation measures with clinical function.

sEMG measure	Clinical measure	*ρ* (95% CI)	*p*	BH-FDR *q*
Panel A. Primary associations (*n* = 110)				
Paretic TA ARV	FMA-LE	0.556 (0.399 to 0.682)	<0.001	<0.001
Paretic TA ARV	BBS	0.492 (0.328 to 0.625)	<0.001	<0.001
Paretic TA ARV	Brunnstrom stage	0.496 (0.326 to 0.636)	<0.001	<0.001
TA paretic/non-paretic ratio	FMA-LE	0.566 (0.422 to 0.682)	<0.001	<0.001
TA paretic/non-paretic ratio	BBS	0.440 (0.275 to 0.583)	<0.001	<0.001
TA paretic/non-paretic ratio	Brunnstrom stage	0.417 (0.262 to 0.564)	<0.001	<0.001
Panel B. Coactivation-FMA-LE associations				
CCI paretic−non-paretic difference	Baseline FMA-LE (*n* = 110)	−0.019 (−0.223 to 0.177)	0.842	0.891
CoA1 paretic−non-paretic difference	Baseline FMA-LE (*n* = 110)	−0.306 (−0.482 to −0.109)	0.001	0.006
Change in CCI paretic−non-paretic difference	Change in FMA-LE (*n* = 78)	−0.023 (−0.272 to 0.226)	0.840	0.840
Change in CoA1 paretic−non-paretic difference	Change in FMA-LE (*n* = 78)	−0.206 (−0.430 to 0.027)	0.070	0.422

Spearman correlations are shown with 95% bootstrap CIs. Panel A includes the six prespecified primary correlations in the baseline sEMG cohort (*n* = 110); *q* values were derived from the complete 36-test baseline sEMG–clinical correlation family and were obtained using the Benjamini–Hochberg false discovery rate correction. For the baseline CCI/CoA1 associations, *q* values were obtained using the Benjamini–Hochberg false discovery rate correction across the complete 36-test baseline sEMG–clinical correlation family. For the longitudinal CCI/CoA1 associations, *q* values were computed within the corresponding six-test change-correlation family. Paretic−non-paretic difference was defined as paretic side minus non-paretic side; delta was defined as discharge minus baseline. TA, tibialis anterior; FMA-LE, Fugl–Meyer Assessment of Lower Extremity; BBS, Berg Balance Scale; CCI, co-contraction index; CoA1, coactivation index 1; CI, confidence interval.

### 3.4 Multivariable associations

After adjustment for age, sex, stroke type, time from stroke onset to baseline assessment, and paretic side, each 10-μV increase in paretic-side TA ARV was associated with an average 0.746-point higher FMA-LE score (95% CI 0.464–1.027) and a 1.262-point higher BBS score (95% CI 0.664–1.860). Each 0.1-unit increase in the TA paretic/non-paretic ratio was associated with an average 1.061-point higher FMA-LE score (95% CI 0.611–1.511) and a 1.646-point higher BBS score (95% CI 0.818–2.473). *p* values from all four HC3 robust models were <0.001 (Table 4).

**Table 4:** Multivariable associations of TA sEMG measures with clinical function.

Outcome	Predictor	Unstandardized *β* (95% CI)	*p*	Adjusted *R*^2^
FMA-LE	Paretic TA ARV, per 10 μV	0.746 (0.464–1.027)	<0.001	0.224
FMA-LE	TA paretic/non-paretic ratio, per 0.1 unit	1.061 (0.611–1.511)	<0.001	0.240
BBS	Paretic TA ARV, per 10 μV	1.262 (0.664–1.860)	<0.001	0.218
BBS	TA paretic/non-paretic ratio, per 0.1 unit	1.646 (0.818–2.473)	<0.001	0.201

Models used the baseline sEMG cohort and were adjusted for age, sex, stroke type, time from stroke onset to baseline assessment, and paretic side. *β* estimates, 95% CIs, and *p* values are from HC3 robust inference. TA, tibialis anterior; sEMG, surface electromyography; FMA-LE, Fugl–Meyer Assessment of Lower Extremity; BBS, Berg Balance Scale; CI, confidence interval.

Exploratory analyses indicated that neither stroke type nor index lesion location significantly modified the associations between the two primary TA measures and clinical outcomes. Time since stroke likewise did not significantly modify the associations of either primary TA measure with the FMA-LE. Significant interactions were observed between baseline Brunnstrom lower-limb stage and both primary TA measures: as baseline Brunnstrom stage increased, the positive associations of paretic-side TA ARV and the TA paretic/non-paretic ratio with the FMA-LE weakened (both interaction *q* = 0.006; Supplementary Tables S4, S5).

### 3.5 Exploratory coactivation analysis

At baseline, the paretic-minus-non-paretic CoA1 difference showed a weak negative correlation with the FMA-LE (*ρ* = −0.306, 95% CI −0.482 to −0.109; BH-FDR *q* = 0.006), whereas the corresponding CCI difference was not associated with the FMA-LE (*ρ* = −0.019, 95% CI −0.223 to 0.177; *q* = 0.891). Longitudinally, the correlation between change in the CoA1 between-side difference and change in the FMA-LE was weak and not statistically significant after multiple-comparison correction (*ρ* = −0.206, 95% CI −0.430 to 0.027; *q* = 0.422). The corresponding CCI correlation was also not significant (*ρ* = −0.023, 95% CI −0.272 to 0.226; *q* = 0.840) (Table 3). Complete longitudinal and sensitivity analyses are provided in Supplementary Table S6.

### 3.6 Longitudinal changes in clinical scales and sEMG

Among the 88 participants with complete longitudinal clinical data, the FMA-LE increased from 23.00 [19.00, 27.25] to 29.50 [23.75, 34.00], with a median within-participant change of 5.00 [1.00, 8.00] (*Z* = 7.485, *q* < 0.001, |*r*| = 0.798). The BBS increased from 19.50 [6.00, 34.25] to 28.00 [13.75, 43.50], with a change of 9.00 [4.00, 12.00] (*Z* = 7.970, *q* < 0.001, |*r*| = 0.850). Brunnstrom lower-limb stage changed from 4.00 [3.00, 4.00] to 4.00 [4.00, 5.00], with a change of 1.00 [0.00, 1.00] (*Z* = 6.737, *q* < 0.001, |*r*| = 0.718) (Table 5; Supplementary Figure S1).

**Table 5:** Longitudinal changes in clinical and selected sEMG outcomes.

Outcome	Baseline, median [IQR]	Discharge, median [IQR]	Individual change, median [IQR]	Wilcoxon *Z*	Raw *p*	BH-FDR *q*	*r*
Clinical outcomes (*n* = 88)							
FMA-LE	23.00 [19.00, 27.25]	29.50 [23.75, 34.00]	5.00 [1.00, 8.00]	7.485	<0.001	<0.001	0.798
BBS	19.50 [6.00, 34.25]	28.00 [13.75, 43.50]	9.00 [4.00, 12.00]	7.970	<0.001	<0.001	0.850
Brunnstrom	4.00 [3.00, 4.00]	4.00 [4.00, 5.00]	1.00 [0.00, 1.00]	6.737	<0.001	<0.001	0.718
Selected sEMG outcomes (*n* = 81)							
Paretic TA ARV (μV)	47.75 [18.72, 97.36]	59.26 [18.74, 98.49]	1.95 [−9.76, 19.65]	1.372	0.170	0.499	0.152
Non-paretic TA ARV (μV)	139.73 [94.61, 178.95]	125.99 [79.51, 159.89]	−12.91 [−56.94, 29.35]	−2.102	0.036	0.213	0.234
TA paretic/non-paretic ratio	0.46 [0.16, 0.80]	0.45 [0.22, 0.91]	0.05 [−0.06, 0.19]	2.291	0.022	0.213	0.255
CCI paretic−non-paretic difference	0.03 [−0.09, 0.15]	0.04 [−0.09, 0.17]	−0.01 [−0.17, 0.21]	0.403	0.687	0.908	0.045
CoA1 paretic−non-paretic difference	5.79 [−7.78, 19.43]	3.74 [−11.92, 19.81]	−7.73 [−33.29, 17.26]	−1.485	0.137	0.499	0.165

Change was defined as discharge minus baseline. Two-sided Wilcoxon signed-rank tests were used. Clinical outcomes were adjusted as a three-test BH-FDR family; sEMG outcomes were adjusted as a 12-test BH-FDR family. Effect size r was calculated as |*Z*|/sqrt(*n*). sEMG, surface electromyography; TA, tibialis anterior; ARV, average rectified value; CCI, co-contraction index; CoA1, coactivation index 1; FMA-LE, Fugl–Meyer Assessment of Lower Extremity; BBS, Berg Balance Scale.

The four-item FMA-LE ankle subscore increased from 4.00 [2.00, 8.00] to 8.00 [4.00, 8.00], with a change of 2.00 [0.00, 4.00] (*Z* = 6.383, *p* < 0.001, |*r*| = 0.680). The number of participants attaining the maximum score of 8 increased from 24/88 (27.3%) at admission to 47/88 (53.4%) at discharge. None of the correlations between change in the ankle subscore and the three prespecified sEMG change measures remained significant after BH-FDR correction (Supplementary Figure S1, Supplementary Table S7).

Among the 81 participants with complete bilateral longitudinal sEMG data, no sEMG measure showed a significant change after BH-FDR correction. The unadjusted *p* value for change in the TA paretic/non-paretic ratio was 0.022, but the adjusted *q* value was 0.213 and was not statistically significant. Selected results are shown in Table 5, and complete results are provided in Supplementary Table S6.

## 4 Discussion

This study showed that task-evoked sEMG amplitudes of the paretic TA and gastrocnemius were lower than those of the non-paretic side during standardized seated active ankle dorsiflexion, with a more pronounced between-side difference for the TA. Paretic-side TA ARV and the TA paretic/non-paretic ratio showed moderate positive correlations with the FMA-LE, BBS, and Brunnstrom lower-limb stage. Both TA measures remained associated with the FMA-LE and BBS after adjustment for age, sex, stroke type, time from stroke onset to baseline assessment, and paretic side. In contrast to these cross-sectional findings, clinical scales improved during inpatient rehabilitation, whereas the primary sEMG measures showed no corresponding significant change after multiple-comparison correction. These findings support the use of TA sEMG as a muscle-specific and task-specific complement to clinical scales, while indicating that cross-sectional functional associations do not necessarily translate into concordant short-term longitudinal changes.

Lower paretic-side TA sEMG amplitude during active ankle dorsiflexion may reflect alterations at several levels of voluntary motor control after stroke. Previous studies have identified impaired motor-unit firing-rate modulation during forceful and rapid ankle dorsiflexion, as well as abnormalities in recruitment, firing behavior, and task endurance during sustained contractions of the paretic dorsiflexors (6, 8). The excitability of spinal motoneurons innervating the TA and other ankle-foot muscles may also change and, together with residual corticospinal drive and reorganization of muscle coordination, influence selective dorsiflexion (7). These neuromuscular abnormalities provide plausible explanations for reduced paretic TA amplitude and its association with clinical function. The more consistent associations of TA measures than gastrocnemius measures with clinical scales may primarily reflect task specificity: the TA is a principal agonist during active ankle dorsiflexion, whereas the gastrocnemius acts mainly as an antagonist and may be more sensitive to co-recruitment and individual movement strategies. Exploratory analyses further indicated that the associations of both primary TA measures with the FMA-LE weakened as baseline Brunnstrom lower-limb stage increased. This finding suggests that the correspondence between TA sEMG and clinical function may vary by stage of motor recovery and requires validation in an independent cohort.

The TA paretic/non-paretic ratio provides within-participant information about bilateral asymmetry beyond absolute paretic-side amplitude and may partly reduce between-participant differences in overall signal scale. However, the non-paretic lower limb is not equivalent to a healthy control limb, because bilateral coordination and task strategies may change after stroke. Both the numerator and denominator are also influenced by signal quality, movement strategy, and repeated electrode placement. The ratio should therefore be interpreted as a measure of bilateral amplitude asymmetry rather than as a fully normalized physiological index of recovery (26). Coactivation analyses showed higher CoA1 on the paretic side and a weak negative correlation between the CoA1 between-side difference and the FMA-LE, whereas CCI did not differ significantly between sides. Higher CoA1 may reflect a greater relative contribution of the gastrocnemius, limited selective motor control, or abnormal co-recruitment during active dorsiflexion. Differences between the CCI and CoA1 findings may arise from their formulas and the signal features they emphasize. Post-stroke co-recruitment is heterogeneous, and commonly used coactivation indices do not consistently correspond to actual trends in joint stiffness (16, 24). CoA1 should therefore be regarded as an exploratory descriptor and cannot be considered superior to CCI on the basis of these findings.

Clinical scales showed large longitudinal improvement effects, with |r| values of 0.798, 0.850, and 0.718 for the FMA-LE, BBS, and Brunnstrom lower-limb stage, respectively. In contrast, the corresponding effect sizes were only 0.152 for paretic-side TA ARV and 0.255 for the TA paretic/non-paretic ratio. Although the unadjusted *p* value for change in the TA ratio was 0.022, the result was not statistically significant after FDR correction (*q* = 0.213). These findings indicate that clinical function improved substantially during the inpatient stay, whereas task-related sEMG measures did not show synchronous and robust longitudinal changes. The four-item FMA-LE ankle subscore also improved, but 53.4% of participants reached the maximum score at discharge, indicating a marked ceiling effect, and change in the subscore was not significantly associated with change in the primary sEMG measures. The discordance between the two types of measures likely reflects differences in the constructs they assess. Clinical scales summarize multijoint control, proximal stability, postural strategies, and task performance, and their change may arise from motor recovery, increased task proficiency, or compensation. The TA measures were obtained from a single seated dorsiflexion task and reflect local, non-MVC-normalized sEMG amplitude and bilateral asymmetry under defined conditions. Previous work likewise indicates that functional improvement may result from recovery or compensation and that post-stroke improvements in walking and clinical function do not necessarily coincide with normalization of lower-limb muscle activation timing or sEMG coordination patterns (27–29).

From a clinical perspective, TA sEMG is best used as complementary information alongside routine scales. Under standardized conditions for seating, initial ankle position, task instruction, electrode placement, and reassessment, paretic-side task-evoked TA sEMG amplitude and the TA paretic/non-paretic ratio can describe local muscle activity and bilateral asymmetry during active dorsiflexion and can be interpreted together with the FMA-LE, BBS, and Brunnstrom stage. When clinical scales improve substantially but TA measures change little, the evaluator may further examine dorsiflexion movement quality, gastrocnemius co-recruitment, and the contribution of other joints and muscle groups. However, the present findings do not support selecting treatment on the basis of a single ARV measurement or using it to predict long-term outcomes. Routine sEMG use remains constrained by electrode placement, repeatability, standardized signal processing, staff training, and workflow integration (13, 14). sEMG alone also cannot provide joint displacement, movement trajectories, or multisegment coordination (30). Because ankle, knee, hip, and trunk kinematics were not recorded concurrently, proximal participation, foot inversion or eversion, and other compensations could not be quantified. Real-time visual feedback and smartphone-based motion capture may provide complementary information on walking coordination and multisegment pelvic and lower-limb movement and could be combined with multimuscle sEMG in future studies (31, 32).

This study has several limitations. First, ARV was not normalized to MVC and may be influenced by electrode position, skin impedance, subcutaneous tissue thickness, task effort, and repeated-measurement conditions. The results therefore represent non-MVC-normalized sEMG amplitude obtained with this device under this task condition and cannot be equated directly with muscle strength, maximal activation capacity, or the degree of neurological recovery. The study also did not provide independent, device-specific validity evidence for the MyoMove-EOW system. Second, seated active ankle dorsiflexion differs from dynamic walking and cannot represent TA activity during swing-phase toe clearance or initial contact. Kinematics were not recorded concurrently, and potential confounders such as ankle contracture, actual range of motion, and subcutaneous fat thickness were not quantitatively controlled. Complete standardized spasticity scale scores were not systematically collected, precluding evaluation or adjustment of the potential influence of spasticity on task-related sEMG measures. Third, no *a priori* sample-size calculation was performed, and only 81 participants contributed longitudinal sEMG data; smaller true effects may therefore have gone undetected. Fourth, this was a single-center observational study of inpatients. The cross-sectional associations cannot establish causality, and exclusion of patients unable to cooperate because of severe cognitive, language, or musculoskeletal comorbidities limits generalizability to the acute phase, community rehabilitation, and more severely affected populations. Patients with and without longitudinal clinical data differed in paretic-side distribution, indicating the possibility of attrition-related selection bias. Finally, the observation period covered only one inpatient rehabilitation stay and could not determine trajectories or prognostic value at 3 or 6 months after discharge.

Future studies should use multicenter prospective designs, prespecify the primary sEMG outcome and follow-up time points before enrollment, and calculate sample size from a clinically meaningful effect. Methodological extensions could include MVC-normalized multimuscle gait-phase sEMG, synchronized video or inertial-sensor kinematics, three-dimensional ankle-foot scanning, and participant-specific finite-element modeling of the ankle and foot to distinguish local recruitment, joint mechanics, and multisegment compensation. Test–retest reliability and longitudinal responsiveness at 3 and 6 months should be evaluated, and real-time TA sEMG biofeedback should be tested as an operational rehabilitation workflow.

## 5 Conclusion

In inpatients undergoing stroke rehabilitation, TA sEMG measures obtained during active ankle dorsiflexion were associated with concurrent lower-limb motor function and balance. Paretic-side TA ARV and the TA paretic/non-paretic ratio may provide muscle-specific and task-specific information complementary to routine clinical scales, but they should not replace the FMA-LE, BBS, or Brunnstrom stage and should not be used alone to judge short-term treatment response or long-term prognosis. Their measurement stability, longitudinal responsiveness, and value within clinical workflows require validation in multicenter studies using standardized methods and longer follow-up.

## Data Availability

The de-identified data supporting the findings of this study are available from the corresponding author upon reasonable request, subject to institutional ethics requirements and patient privacy restrictions.

## References

[1] National Institute for Health and Care Excellence Stroke Rehabilitation in Adults. NICE Guideline NG236 London NICE (2023). Available online at: https://www.nice.org.uk/guidance/ng236 (accessed August 2, 2026).

[2] Winstein CJ Stein J Arena R Bates B Cherney LR Cramer SC . Guidelines for adult stroke rehabilitation and recovery: a guideline for healthcare professionals from the American Heart Association/American Stroke Association. Stroke. (2016) 47:e98–e169. doi: 10.1161/STR.0000000000000098, 27145936

[3] Yoo YJ Lim SH. Assessment of lower limb motor function, ambulation, and balance after stroke. Brain Neurorehabil. (2022) 15:e17. doi: 10.12786/bn.2022.15.e17, 36743203 PMC9833471

[4] Srivastava S Kindred JH Seamon BA Charalambous CC Boan AD Kautz SA . A novel biomechanical indicator for impaired ankle dorsiflexion function during walking in individuals with chronic stroke. Gait Posture. (2024) 107:246–52. doi: 10.1016/j.gaitpost.2023.10.012, 37923642 PMC11730035

[5] Dorsch S Ada L Canning CG Al-Zharani M Dean C. The strength of the ankle dorsiflexors has a significant contribution to walking speed in people who can walk independently after stroke: an observational study. Arch Phys Med Rehabil. (2012) 93:1072–6. doi: 10.1016/j.apmr.2012.01.005, 22464738

[6] Chou LW Palmer JA Binder-Macleod SA Knight CA. Motor unit rate coding is severely impaired during forceful and fast muscular contractions in individuals post stroke. J Neurophysiol. (2013) 109:2947–54. doi: 10.1152/jn.00615.2012, 23554434 PMC3680820

[7] Liu G Chia CH Cao Y Tang XW Tian S Shen XY . Differential changed excitability of spinal motor neurons innervating tibialis anterior and peroneus muscles cause foot inversion after stroke. Front Neurol. (2020) 11:544912. doi: 10.3389/fneur.2020.544912, 33329299 PMC7732441

[8] Negro F Bathon KE Nguyen JN Bannon CG Orizio C Hunter SK . Impaired firing behavior of individually tracked paretic motor units during fatiguing contractions of the dorsiflexors and functional implications post stroke. Front Neurol. (2020) 11:540893. doi: 10.3389/fneur.2020.540893, 33192970 PMC7658471

[9] Van Criekinge T Heremans C Burridge J Deutsch JE Hammerbeck U Hollands K . Standardized measurement of balance and mobility post-stroke: consensus-based core recommendations from the third stroke recovery and rehabilitation roundtable. Neurorehabil Neural Repair. (2024) 38:41–51. doi: 10.1177/15459683231209154, 37837351

[10] Brunnstrom S. Movement Therapy in Hemiplegia: A Neurophysiological Approach. New York: Harper & Row (1970).

[11] Fugl-Meyer AR Jääskö L Leyman I Olsson S Steglind S. The post-stroke hemiplegic patient. 1. A method for evaluation of physical performance. Scand J Rehabil Med. (1975) 7:13–31. doi: 10.2340/1650197771331, 1135616

[12] Blum L Korner-Bitensky N. Usefulness of the berg balance scale in stroke rehabilitation: a systematic review. Phys Ther. (2008) 88:559–66. doi: 10.2522/ptj.20070205, 18292215

[13] Campanini I Disselhorst-Klug C Rymer WZ Merletti R. Surface EMG in clinical assessment and neurorehabilitation: barriers limiting its use. Front Neurol. (2020) 11:934. doi: 10.3389/fneur.2020.00934, 32982942 PMC7492208

[14] Hur Y Oh BM Seo HG Hyun SE Kim DJ Kim H . Reliability of surface electromyography from the lower-limb muscles during maximal and submaximal voluntary isometric contractions in in-bed healthy individuals and patients with subacute stroke. Brain Neurorehabil. (2024) 17:e14. doi: 10.12786/bn.2024.17.e14, 39113922 PMC11300959

[15] Meng L Zhang T Zhao X Wang D Xu R Yang A . A quantitative lower limb function assessment method based on fusion of surface EMG and inertial data in stroke patients during cycling task. Biomed Signal Process Control. (2023) 85:104880. doi: 10.1016/j.bspc.2023.104880

[16] Banks CL Huang HJ Little VL Patten C. Electromyography exposes heterogeneity in muscle co-contraction following stroke. Front Neurol. (2017) 8:699. doi: 10.3389/fneur.2017.00699, 29312124 PMC5743661

[17] Androwis GJ Pilkar R Ramanujam A Nolan KJ. Electromyography assessment during gait in a robotic exoskeleton for acute stroke. Front Neurol. (2018) 9:630. doi: 10.3389/fneur.2018.00630, 30131756 PMC6090052

[18] Ye S Tao L Gong S Ma Y Wu J Li W . Upper limb motor assessment for stroke with force, muscle activation and interhemispheric balance indices based on sEMG and fNIRS. Front Neurol. (2024) 15:1337230. doi: 10.3389/fneur.2024.1337230, 38694770 PMC11061400

[19] Xu Y Wang J Wang S Li J Hou Y Guo A. Neuromuscular conditions in post-stroke ankle-foot dysfunction reflected by surface electromyography. J Neuroeng Rehabil. (2024) 21:137. doi: 10.1186/s12984-024-01435-5, 39107804 PMC11304728

[20] Noor A Waris A Gilani SO Kashif AS Jochumsen M Iqbal J . Decoding of ankle joint movements in stroke patients using surface electromyography. Sensors. (2021) 21:1575. doi: 10.3390/s21051575, 33668229 PMC7956677

[21] von Elm E Altman DG Egger M Pocock SJ Gøtzsche PC Vandenbroucke JP . The strengthening the reporting of observational studies in epidemiology (STROBE) statement: guidelines for reporting observational studies. PLoS Med. (2007) 4:e296. doi: 10.1371/journal.pmed.0040296, 17941714 PMC2020495

[22] Hermens HJ Freriks B Disselhorst-Klug C Rau G. Development of recommendations for SEMG sensors and sensor placement procedures. J Electromyogr Kinesiol. (2000) 10:361–74. doi: 10.1016/S1050-6411(00)00027-4, 11018445

[23] Falconer K Winter DA. Quantitative assessment of co-contraction at the ankle joint in walking. Electromyogr Clin Neurophysiol. (1985) 25:135–49. 3987606

[24] Li G Shourijeh MS Ao D Patten C Fregly BJ. How well do commonly used co-contraction indices approximate lower limb joint stiffness trends during gait for individuals post-stroke? Front Bioeng Biotechnol. (2021) 8:588908. doi: 10.3389/fbioe.2020.588908, 33490046 PMC7817819

[25] Gagnat Y Brændvik SM Roeleveld K. Surface electromyography normalization affects the interpretation of muscle activity and coactivation in children with cerebral palsy during walking. Front Neurol. (2020) 11:202. doi: 10.3389/fneur.2020.00202, 32362862 PMC7180206

[26] Menezes KKP Nascimento LR Pinheiro MB Scianni AA Faria CDCM Avelino PR . Lower-limb motor coordination is significantly impaired in ambulatory people with chronic stroke: a cross-sectional study. J Rehabil Med. (2017) 49:322–6. doi: 10.2340/16501977-2215, 28352935

[27] Levin MF Kleim JA Wolf SL. What do motor recovery and compensation mean in patients following stroke? Neurorehabil Neural Repair. (2009) 23:313–9. doi: 10.1177/1545968308328727, 19118128

[28] Den Otter AR Geurts ACH Mulder T Duysens J. Gait recovery is not associated with changes in the temporal patterning of muscle activity during treadmill walking in patients with post-stroke hemiparesis. Clin Neurophysiol. (2006) 117:4–15. doi: 10.1016/j.clinph.2005.08.014, 16337186

[29] Buurke JH Nene AV Kwakkel G Erren-Wolters V IJzerman MJ Hermens HJ. Recovery of gait after stroke: what changes? Neurorehabil Neural Repair. (2008) 22:676–83. doi: 10.1177/1545968308317972, 18971383

[30] Disselhorst-Klug C Williams S. Surface electromyography meets biomechanics: correct interpretation of sEMG-signals in neuro-rehabilitation needs biomechanical input. Front Neurol. (2020) 11:603550. doi: 10.3389/fneur.2020.603550, 33424754 PMC7793912

[31] Liu LY Sangani S Patterson KK Fung J Lamontagne A. Instantaneous effect of real-time avatar visual feedback on interlimb coordination during walking post-stroke. Clin Biomech. (2022) 100:105821. doi: 10.1016/j.clinbiomech.2022.105821, 36435074

[32] Peng Y Wang W Zeng Y Chen Z Li H Li G. Analysis of pelvis and lower limb coordination in stroke patients using smartphone-based motion capture. IEEE Trans Biomed Eng. (2025) 72:2425–36. doi: 10.1109/TBME.2025.3543346, 40036460

