# Supplementary material for: Tibialis anterior surface electromyography during ankle dorsiflexion as a complementary assessment measure after stroke
Source: Front Neurol. 2026 Sep 10;17:1913483. doi: 10.3389/fneur.2026.1913483 (PMC13604026; doi:10.3389/fneur.2026.1913483)
Supplement: Supplementary file 1 [file Data_Sheet_1.PDF]

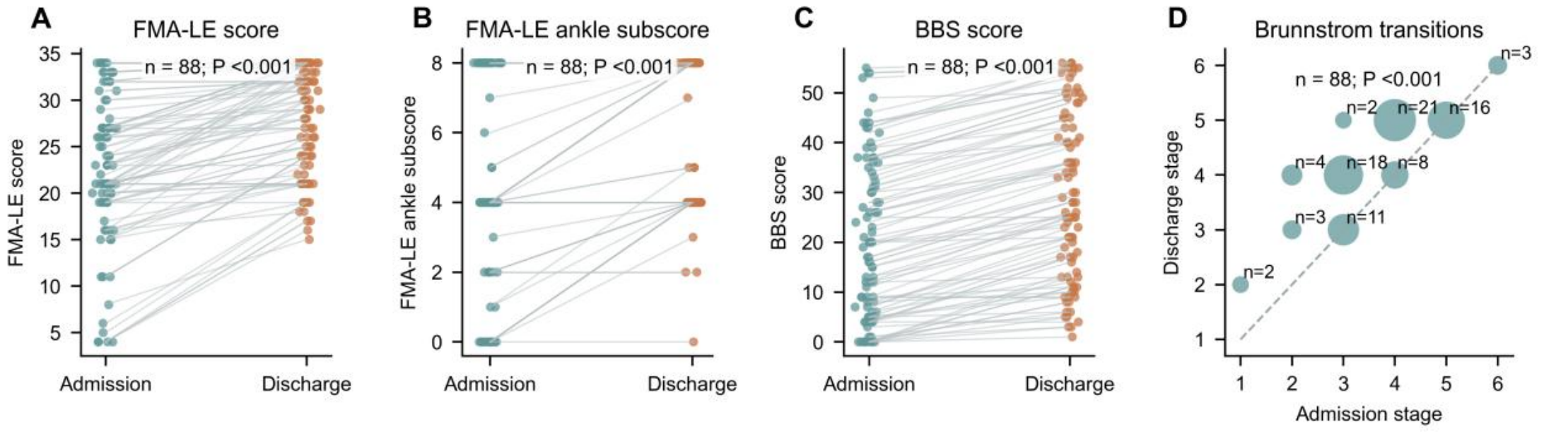

Supplementary Figure S1. Admission-to-discharge changes in clinical outcomes (n=88).

(A) FMA-LE total score; (B) four-item FMA-LE ankle subscore; (C) BBS score; and (D) Brunnstrom lower-limb stage transitions. In panels A–C, lines connect paired admission and discharge observations from the same participant. In panel D, bubble size and labels indicate the number of participants for each admission-to-discharge stage transition; the dashed diagonal indicates no change. P values are from two-sided Wilcoxon signed-rank tests. FMA-LE, Fugl–Meyer Assessment of Lower Extremity; BBS, Berg Balance Scale.
