# Supplementary material for: Tibialis anterior surface electromyography during ankle dorsiflexion as a complementary assessment measure after stroke
Source: Front Neurol. 2026 Sep 10;17:1913483. doi: 10.3389/fneur.2026.1913483 (PMC13604026; doi:10.3389/fneur.2026.1913483)
Supplement: Supplementary file 2 [file Table_1.DOCX]

**Supplementary Table S1. Baseline characteristics according to availability of longitudinal clinical and sEMG data in the baseline sEMG cohort (n=110).**

| **Panel A. Longitudinal clinical data availability** |  |  |  |  |
| --- | --- | --- | --- | --- |
| **Variable** | **Longitudinal clinical data available (n=88)** | **Longitudinal clinical data unavailable (n=22)** | **P value** | **Effect size** |
| Age, years | 61.00 [53.75, 70.00] | 66.50 [59.25, 70.75] | 0.060 | r=-0.260 |
| Time from stroke onset, days | 26.50 [12.75, 62.50] | 28.00 [18.00, 50.25] | 0.991 | r=-0.002 |
| Baseline FMA-LE | 23.00 [19.00, 27.25] | 23.00 [21.00, 27.00] | 0.793 | r=-0.037 |
| Baseline BBS | 19.50 [6.00, 34.25] | 13.50 [6.00, 24.50] | 0.352 | r=0.129 |
| Paretic side, n (%) |  |  | 0.003 | OR=4.829 (95% CI 1.595 to 16.674) |
| Left | 57 (64.8%) | 6 (27.3%) |  |  |
| Right | 31 (35.2%) | 16 (72.7%) |  |  |
| **Panel B. Longitudinal sEMG data availability** |  |  |  |  |
| **Variable** | **Longitudinal sEMG data available (n=81)** | **Longitudinal sEMG data unavailable (n=29)** | **P value** | **Effect size** |
| Age, years | 61.00 [54.00, 69.00] | 67.00 [58.00, 71.00] | 0.052 | r=-0.244 |
| Time from stroke onset, days | 28.00 [13.00, 62.00] | 24.00 [15.00, 51.00] | 0.760 | r=0.039 |
| Baseline FMA-LE | 23.00 [19.00, 27.00] | 21.00 [21.00, 27.00] | 0.809 | r=-0.031 |
| Baseline BBS | 20.00 [7.00, 36.00] | 12.00 [6.00, 25.00] | 0.102 | r=0.205 |
| Paretic side, n (%) |  |  | 0.051 | OR=2.388 (95% CI 0.931 to 6.313) |
| Left | 51 (63.0%) | 12 (41.4%) |  |  |
| Right | 30 (37.0%) | 17 (58.6%) |  |  |

Note. Continuous values are medians [IQR] and were compared using Mann-Whitney U tests, with rank-biserial r as the effect size. P values, odds ratios, and 95% confidence intervals for paretic-side distributions were obtained using Fisher's exact method. The odds ratios compare left versus right paretic side, with right paretic side as the reference. sEMG, surface electromyography; FMA-LE, Fugl-Meyer Assessment of Lower Extremity; BBS, Berg Balance Scale; OR, odds ratio; CI, confidence interval.

**Supplementary Table S2. Complete baseline paretic−non-paretic comparisons for TA and GAS measures.**

| **Measure** | **Paretic side, median [IQR]** | **Non-paretic side, median [IQR]** | **Paretic−non-paretic difference, median [IQR]** | **Paretic/non-paretic ratio, median [IQR]** | **Z** | **Raw P** | **BH-FDR q** | **r** |
| --- | --- | --- | --- | --- | --- | --- | --- | --- |
| **Panel A. ARV and iEMG measures (n=110)** |  |  |  |  |  |  |  |  |
| TA ARV, µV | 48.69 [23.98, 97.13] | 139.26 [95.25, 179.24] | −59.75 [−122.68, −24.49] | 0.46 [0.19, 0.80] | −8.441 | <0.001 | <0.001 | 0.805 |
| GAS ARV, µV | 25.89 [13.72, 52.55] | 48.86 [33.29, 77.54] | −21.24 [−44.95, −2.36] | 0.53 [0.24, 0.91] | −5.092 | <0.001 | <0.001 | 0.486 |
| TA iEMG, µV*s | 2435.39 [926.55, 3858.90] | 5274.61 [3655.26, 7166.65] | −2347.95 [−4831.33, −632.85] | 0.46 [0.19, 0.83] | −7.377 | <0.001 | <0.001 | 0.703 |
| GAS iEMG, µV*s | 1313.39 [601.55, 2354.71] | 2191.86 [1462.85, 3372.62] | −805.83 [−1770.47, 101.50] | 0.55 [0.25, 1.06] | −3.902 | <0.001 | <0.001 | 0.372 |
| **Panel B. CCI and CoA1 measures (n=110)** |  |  |  |  |  |  |  |  |
| CCI, unitless | 0.52 [0.41, 0.66] | 0.50 [0.38, 0.64] | 0.02 [−0.08, 0.14] |  | 1.382 | 0.167 | 0.167 | 0.132 |
| CoA1, % | 61.73 [45.18, 109.85] | 55.04 [43.00, 87.49] | 6.25 [−6.95, 34.02] |  | 2.969 | 0.003 | 0.004 | 0.283 |

Note. Values are medians [IQR]. Paretic−non-paretic difference was defined as paretic side minus non-paretic side. Two-sided Wilcoxon signed-rank tests were used; q values were computed using BH-FDR across the six comparisons. r=|Z|/sqrt(n). TA, tibialis anterior; GAS, gastrocnemius; ARV, average rectified value; iEMG, integrated electromyography; CCI, co-contraction index; CoA1, coactivation index 1.

**Supplementary Table S3. Cross-sectional associations of baseline sEMG measures with FMA-LE, BBS, and Brunnstrom stage (n=110).**

| **sEMG measure** | **FMA-LE ρ (95% CI)** | **FMA-LE P** | **FMA-LE q** | **BBS ρ (95% CI)** | **BBS P** | **BBS q** | **Brunnstrom ρ (95% CI)** | **Brunnstrom P** | **Brunnstrom q** |
| --- | --- | --- | --- | --- | --- | --- | --- | --- | --- |
| Paretic TA ARV | 0.556 (0.399 to 0.682) | <0.001 | <0.001 | 0.492 (0.328 to 0.625) | <0.001 | <0.001 | 0.496 (0.326 to 0.636) | <0.001 | <0.001 |
| Non-paretic TA ARV | 0.029 (−0.166 to 0.225) | 0.767 | 0.891 | 0.108 (−0.101 to 0.303) | 0.262 | 0.336 | 0.124 (−0.067 to 0.312) | 0.196 | 0.272 |
| TA paretic/non-paretic ratio | 0.566 (0.422 to 0.682) | <0.001 | <0.001 | 0.440 (0.275 to 0.583) | <0.001 | <0.001 | 0.417 (0.262 to 0.564) | <0.001 | <0.001 |
| Paretic GAS ARV | 0.303 (0.106 to 0.490) | 0.001 | 0.006 | 0.239 (0.064 to 0.402) | 0.012 | 0.042 | 0.273 (0.092 to 0.450) | 0.004 | 0.015 |
| Non-paretic GAS ARV | 0.119 (−0.068 to 0.307) | 0.217 | 0.289 | 0.007 (−0.175 to 0.194) | 0.940 | 0.967 | 0.022 (−0.162 to 0.216) | 0.818 | 0.891 |
| GAS paretic/non-paretic ratio | 0.137 (−0.076 to 0.340) | 0.154 | 0.251 | 0.223 (0.045 to 0.390) | 0.019 | 0.058 | 0.237 (0.048 to 0.412) | 0.013 | 0.042 |
| Paretic CCI | −0.133 (−0.328 to 0.069) | 0.166 | 0.259 | −0.131 (−0.319 to 0.052) | 0.173 | 0.259 | −0.128 (−0.326 to 0.072) | 0.183 | 0.263 |
| Non-paretic CCI | −0.187 (−0.374 to 0.005) | 0.051 | 0.107 | −0.205 (−0.383 to −0.004) | 0.031 | 0.080 | −0.198 (−0.368 to −0.008) | 0.038 | 0.092 |
| CCI paretic−non-paretic difference | −0.019 (−0.223 to 0.177) | 0.842 | 0.891 | 0.024 (−0.176 to 0.223) | 0.800 | 0.891 | 0.061 (−0.133 to 0.253) | 0.530 | 0.636 |
| Paretic CoA1 | −0.183 (−0.376 to 0.024) | 0.056 | 0.112 | −0.214 (−0.383 to −0.021) | 0.025 | 0.069 | −0.189 (−0.376 to 0.011) | 0.048 | 0.107 |
| Non-paretic CoA1 | −0.002 (−0.207 to 0.200) | 0.985 | 0.985 | −0.160 (−0.340 to 0.030) | 0.096 | 0.181 | −0.141 (−0.334 to 0.054) | 0.143 | 0.245 |
| CoA1 paretic−non-paretic difference | −0.306 (−0.482 to −0.109) | 0.001 | 0.006 | −0.157 (−0.345 to 0.042) | 0.102 | 0.184 | −0.096 (−0.292 to 0.103) | 0.316 | 0.392 |

Note. Spearman correlations are shown with 95% bootstrap CIs. q values were computed using BH-FDR across the complete 36-test family. Paretic−non-paretic difference was defined as paretic side minus non-paretic side. sEMG, surface electromyography; FMA-LE, Fugl-Meyer Assessment of Lower Extremity; BBS, Berg Balance Scale; CI, confidence interval.

**Supplementary Table S4. Exploratory stratified analyses by stroke type and index lesion location.**

| **Predictor** | **Outcome** | **Group 1 ρ (95% CI); P** | **Group 2 ρ (95% CI); P** | **Interaction β (group 2 vs group 1; 95% CI)** | **Interaction P** | **Interaction q** |
| --- | --- | --- | --- | --- | --- | --- |
| **Panel A. Stroke type: ischemic (n=88) vs hemorrhagic (n=22)** |  |  |  |  |  |  |
| Paretic-side TA ARV (per 10 µV) | FMA-LE | 0.483 (0.304 to 0.629); P<0.001 | 0.685 (0.370 to 0.859); P<0.001 | 0.397 (−0.226 to 1.020) | 0.209 | 0.538 |
| Paretic-side TA ARV (per 10 µV) | BBS | 0.475 (0.294 to 0.622); P<0.001 | 0.328 (−0.109 to 0.658); P=0.137 | −0.532 (−2.511 to 1.447) | 0.595 | 0.714 |
| Paretic-side TA ARV (per 10 µV) | Brunnstrom stage | 0.516 (0.344 to 0.655); P<0.001 | 0.208 (−0.234 to 0.579); P=0.353 | −0.085 (−0.199 to 0.029) | 0.141 | 0.538 |
| TA paretic/non-paretic ratio (per 0.1-unit increase) | FMA-LE | 0.547 (0.381 to 0.679); P<0.001 | 0.604 (0.245 to 0.818); P=0.003 | 0.366 (−0.777 to 1.509) | 0.527 | 0.703 |
| TA paretic/non-paretic ratio (per 0.1-unit increase) | BBS | 0.472 (0.291 to 0.620); P<0.001 | 0.143 (−0.297 to 0.532); P=0.526 | −0.970 (−3.276 to 1.337) | 0.406 | 0.696 |
| TA paretic/non-paretic ratio (per 0.1-unit increase) | Brunnstrom stage | 0.445 (0.260 to 0.599); P<0.001 | 0.158 (−0.283 to 0.543); P=0.483 | −0.074 (−0.207 to 0.058) | 0.269 | 0.538 |
| **Panel B. Index lesion location: supratentorial (n=79) vs infratentorial (n=31)** |  |  |  |  |  |  |
| Paretic-side TA ARV (per 10 µV) | FMA-LE | 0.592 (0.427 to 0.719); P<0.001 | 0.423 (0.081 to 0.676); P=0.018 | −0.238 (−0.908 to 0.432) | 0.483 | 0.703 |
| Paretic-side TA ARV (per 10 µV) | BBS | 0.554 (0.379 to 0.690); P<0.001 | 0.248 (−0.116 to 0.554); P=0.178 | −0.943 (−2.429 to 0.543) | 0.211 | 0.538 |
| Paretic-side TA ARV (per 10 µV) | Brunnstrom stage | 0.576 (0.407 to 0.707); P<0.001 | 0.281 (−0.081 to 0.578); P=0.125 | −0.059 (−0.157 to 0.039) | 0.235 | 0.538 |
| TA paretic/non-paretic ratio (per 0.1-unit increase) | FMA-LE | 0.571 (0.401 to 0.704); P<0.001 | 0.583 (0.289 to 0.777); P<0.001 | −0.090 (−0.966 to 0.787) | 0.839 | 0.839 |
| TA paretic/non-paretic ratio (per 0.1-unit increase) | BBS | 0.494 (0.306 to 0.645); P<0.001 | 0.242 (−0.123 to 0.549); P=0.190 | −0.391 (−2.356 to 1.573) | 0.694 | 0.757 |
| TA paretic/non-paretic ratio (per 0.1-unit increase) | Brunnstrom stage | 0.558 (0.384 to 0.694); P<0.001 | 0.061 (−0.300 to 0.406); P=0.746 | −0.134 (−0.266 to −0.002) | 0.046 | 0.538 |

Note. Within-subgroup estimates are Spearman ρ with 95% CIs. Interaction estimates are HC3 robust β terms from formal interaction models and are reported per 10 µV for paretic-side TA ARV and per 0.1-unit increase for TA paretic/non-paretic ratio. q values were computed within the interaction family. TA, tibialis anterior; FMA-LE, Fugl-Meyer Assessment of Lower Extremity; BBS, Berg Balance Scale; CI, confidence interval.

**Supplementary Table S5. Exploratory analyses by time since stroke, baseline severity, formal interactions, and fixed-sample-size sensitivity.**

| **Analysis** | **Predictor** | **Outcome** | **≤30 days (n=58) / I–III (n=48)** | **31–90 days (n=38) / IV (n=38)** | **>90 days (n=14) / V–VI (n=24)** | **P or interaction P** | **q** |
| --- | --- | --- | --- | --- | --- | --- | --- |
| **Panel A. Time since stroke strata** |  |  |  |  |  |  |  |
| Time since stroke | Paretic TA ARV | FMA-LE | 0.571 (0.367 to 0.723); P<0.001 | 0.511 (0.228 to 0.714); P=0.001 | 0.650 (0.183 to 0.878); P=0.012 |  |  |
| Time since stroke | Paretic TA ARV | BBS | 0.447 (0.214 to 0.633); P<0.001 | 0.495 (0.208 to 0.703); P=0.002 | 0.520 (−0.015 to 0.823); P=0.057 |  |  |
| Time since stroke | Paretic TA ARV | Brunnstrom stage | 0.569 (0.365 to 0.722); P<0.001 | 0.337 (0.019 to 0.593); P=0.039 | 0.605 (0.109 to 0.859); P=0.022 |  |  |
| Time since stroke | TA paretic/non-paretic ratio | FMA-LE | 0.596 (0.399 to 0.740); P<0.001 | 0.494 (0.207 to 0.703); P=0.002 | 0.650 (0.183 to 0.878); P=0.012 |  |  |
| Time since stroke | TA paretic/non-paretic ratio | BBS | 0.393 (0.150 to 0.592); P=0.002 | 0.486 (0.196 to 0.697); P=0.002 | 0.511 (−0.027 to 0.819); P=0.062 |  |  |
| Time since stroke | TA paretic/non-paretic ratio | Brunnstrom stage | 0.446 (0.213 to 0.632); P<0.001 | 0.394 (0.085 to 0.634); P=0.014 | 0.415 (−0.149 to 0.775); P=0.141 |  |  |
| **Panel B. Baseline severity strata** |  |  |  |  |  |  |  |
| Baseline severity | Paretic TA ARV | FMA-LE | 0.628 (0.418 to 0.774); P<0.001 | 0.232 (−0.095 to 0.513); P=0.162 | 0.041 (−0.369 to 0.437); P=0.850 |  |  |
| Baseline severity | Paretic TA ARV | BBS | 0.276 (−0.009 to 0.519); P=0.058 | 0.303 (−0.019 to 0.568); P=0.064 | −0.102 (−0.485 to 0.314); P=0.635 |  |  |
| Baseline severity | Paretic TA ARV | Brunnstrom stage | 0.312 (0.030 to 0.548); P=0.031 | NE | 0.173 (−0.248 to 0.539); P=0.419 |  |  |
| Baseline severity | TA paretic/non-paretic ratio | FMA-LE | 0.643 (0.439 to 0.784); P<0.001 | 0.213 (−0.114 to 0.499); P=0.199 | 0.365 (−0.045 to 0.670); P=0.080 |  |  |
| Baseline severity | TA paretic/non-paretic ratio | BBS | 0.107 (−0.182 to 0.380); P=0.468 | 0.337 (0.020 to 0.593); P=0.038 | 0.186 (−0.235 to 0.548); P=0.384 |  |  |
| Baseline severity | TA paretic/non-paretic ratio | Brunnstrom stage | 0.256 (−0.031 to 0.503); P=0.079 | NE | −0.009 (−0.411 to 0.396); P=0.966 |  |  |
| **Panel C. Formal interaction tests** |  |  |  |  |  |  |  |
| Continuous time since stroke interaction | Paretic-side TA ARV (per 10 µV) | FMA-LE | −0.089 (−0.374 to 0.197) |  |  | 0.539 | 0.662 |
| Continuous time since stroke interaction | TA paretic/non-paretic ratio (per 0.1-unit increase) | FMA-LE | −0.350 (−0.780 to 0.081) |  |  | 0.110 | 0.330 |
| Baseline severity interaction | Paretic-side TA ARV (per 10 µV) | FMA-LE | −0.339 (−0.538 to −0.141) |  |  | 0.001 | 0.006 |
| Baseline severity interaction | TA paretic/non-paretic ratio (per 0.1-unit increase) | FMA-LE | −0.497 (−0.787 to −0.206) |  |  | <0.001 | 0.006 |
| **Panel D. Fixed-sample-size sensitivity analyses** |  |  |  |  |  |  |  |
| Fixed-sample-size sensitivity | Baseline correlations |  | n=110 | minimum detectable \|rho\|=0.264 | alpha=0.05, power=0.80 |  |  |
| Fixed-sample-size sensitivity | Clinical paired change |  | n=88 | minimum detectable dz=0.302 | alpha=0.05, power=0.80 |  |  |
| Fixed-sample-size sensitivity | sEMG paired change |  | n=81 | minimum detectable dz=0.315 | alpha=0.05, power=0.80 |  |  |

Note. Time since stroke was grouped as ≤30 days, 31–90 days, and >90 days. Baseline severity was grouped as Brunnstrom I–III, IV, and V–VI. Within-stratum values are Spearman ρ (95% CI) with P values. Formal interactions used HC3 robust models and are reported per 10 µV for paretic-side TA ARV and per 0.1-unit increase for TA paretic/non-paretic ratio. NE, not estimable; correlations with baseline Brunnstrom stage were not estimable in the Brunnstrom IV stratum because the stratifying outcome was constant. Panel D reports design sensitivity calculations under two-sided α=0.05 and 80% power; n is retained because it is the target of the sensitivity analysis. TA, tibialis anterior; FMA-LE, Fugl-Meyer Assessment of Lower Extremity; BBS, Berg Balance Scale; CI, confidence interval.

**Supplementary Table S6. Longitudinal sEMG changes, CCI/CoA1 change correlations, and CoA1 outlier sensitivity analyses.**

| **Outcome or association** | **Baseline** | **Discharge** | **Change or ρ (95% CI)** | **Z** | **Raw P** | **BH-FDR q** | **r** |
| --- | --- | --- | --- | --- | --- | --- | --- |
| **Panel A. Longitudinal changes in all 12 sEMG measures (n=81)** |  |  |  |  |  |  |  |
| Paretic TA ARV (µV) | 47.75 [18.72, 97.36] | 59.26 [18.74, 98.49] | 1.95 [−9.76, 19.65] | 1.372 | 0.170 | 0.499 | 0.152 |
| Non-paretic TA ARV (µV) | 139.73 [94.61, 178.95] | 125.99 [79.51, 159.89] | −12.91 [−56.94, 29.35] | −2.102 | 0.036 | 0.213 | 0.234 |
| TA paretic/non-paretic ratio | 0.46 [0.16, 0.80] | 0.45 [0.22, 0.91] | 0.05 [−0.06, 0.19] | 2.291 | 0.022 | 0.213 | 0.255 |
| Paretic GAS ARV (µV) | 25.83 [13.28, 49.77] | 23.88 [12.58, 43.16] | 0.36 [−15.06, 10.18] | 0.210 | 0.834 | 0.908 | 0.023 |
| Non-paretic GAS ARV (µV) | 50.51 [34.58, 77.48] | 43.26 [33.67, 59.61] | −2.62 [−23.38, 14.34] | −0.864 | 0.388 | 0.775 | 0.096 |
| GAS paretic/non-paretic ratio | 0.47 [0.22, 0.88] | 0.41 [0.22, 0.96] | 0.05 [−0.23, 0.28] | 0.436 | 0.663 | 0.908 | 0.048 |
| Paretic CCI | 0.50 [0.39, 0.74] | 0.51 [0.39, 0.67] | −0.03 [−0.21, 0.13] | −0.525 | 0.600 | 0.908 | 0.058 |
| Non-paretic CCI | 0.47 [0.37, 0.63] | 0.47 [0.39, 0.56] | 0.01 [−0.14, 0.12] | −0.252 | 0.801 | 0.908 | 0.028 |
| CCI paretic−non-paretic difference | 0.03 [-0.09, 0.15] | 0.04 [-0.09, 0.17] | −0.01 [−0.17, 0.21] | 0.403 | 0.687 | 0.908 | 0.045 |
| Paretic CoA1 (%) | 61.59 [43.87, 110.31] | 58.94 [42.83, 94.09] | −8.56 [−29.71, 16.93] | −1.259 | 0.208 | 0.499 | 0.140 |
| Non-paretic CoA1 (%) | 54.92 [42.34, 86.13] | 52.03 [41.97, 76.73] | 1.34 [−18.91, 14.08] | 0.115 | 0.908 | 0.908 | 0.013 |
| CoA1 paretic−non-paretic difference | 5.79 [-7.78, 19.43] | 3.74 [-11.92, 19.81] | −7.73 [−33.29, 17.26] | −1.485 | 0.137 | 0.499 | 0.165 |
| **Panel B. Associations of changes in CCI/CoA1 with clinical changes** |  |  |  |  |  |  |  |
| Change in CCI paretic−non-paretic difference with Change in FMA-LE | n=78 |  | −0.023 (−0.272 to 0.226) |  | 0.840 | 0.840 |  |
| Change in CCI paretic−non-paretic difference with Change in BBS | n=78 |  | −0.040 (−0.265 to 0.191) |  | 0.728 | 0.840 |  |
| Change in CCI paretic−non-paretic difference with Change in Brunnstrom stage | n=81 |  | −0.095 (−0.320 to 0.135) |  | 0.401 | 0.840 |  |
| Change in CoA1 paretic−non-paretic difference with Change in FMA-LE | n=78 |  | −0.206 (−0.430 to 0.027) |  | 0.070 | 0.422 |  |
| Change in CoA1 paretic−non-paretic difference with Change in BBS | n=78 |  | 0.055 (−0.158 to 0.261) |  | 0.632 | 0.840 |  |
| Change in CoA1 paretic−non-paretic difference with Change in Brunnstrom stage | n=81 |  | −0.042 (−0.268 to 0.191) |  | 0.709 | 0.840 |  |
| **Panel C. CoA1 outlier sensitivity analyses** |  |  |  |  |  |  |  |
| Baseline CoA1 paretic−non-paretic difference with FMA-LE, all cases | n=110 |  | −0.306 (−0.482 to −0.109) |  | 0.001 | 0.006 |  |
| Baseline CoA1 paretic−non-paretic difference with FMA-LE, excluding Tukey extremes | n=100 |  | −0.277 (−0.472 to −0.065) |  | 0.005 |  |  |
| Change in CoA1 paretic−non-paretic difference with Change in FMA-LE, excluding Tukey extremes | n=69 |  | −0.147 (−0.385 to 0.104) |  | 0.229 |  |  |

Note. Change was defined as discharge minus baseline. Panel A used two-sided Wilcoxon signed-rank tests with BH-FDR q values across 12 sEMG measures. Panels B and C used Spearman correlations; n is shown where the available sample differed by analysis. sEMG, surface electromyography; CCI, co-contraction index; CoA1, coactivation index 1; CI, confidence interval.

**Supplementary Table S7. Exploratory longitudinal analysis and ceiling effect of the four-item FMA-LE ankle subscore.**

| **Analysis** | **n** | **Baseline or ρ (95% CI)** | **Discharge** | **Change** | **P** | **q** | **r** |
| --- | --- | --- | --- | --- | --- | --- | --- |
| **Panel A. FMA-LE ankle 4-item subscore change** | **88** | **4.00 [2.00, 8.00]** | **8.00 [4.00, 8.00]** | **2.00 [0.00, 4.00]; Z=6.383** | **<0.001** | **—** | **0.680** |
| **Panel B. Ceiling effect** |  |  |  |  |  |  |  |
| Baseline ceiling at maximum score of 8 | 88 | 24 (27.3%) |  |  |  |  |  |
| Discharge ceiling at maximum score of 8 | 88 |  | 47 (53.4%) |  |  |  |  |
| **Panel C. Associations between ankle subscore change and sEMG changes** |  |  |  |  |  |  |  |
| Delta paretic TA ARV with ankle subscore change | 78 | −0.202 (−0.418 to 0.020) |  |  | 0.076 | 0.203 |  |
| Delta TA ratio with ankle subscore change | 78 | −0.023 (−0.263 to 0.214) |  |  | 0.840 | 0.840 |  |
| Change in CoA1 paretic−non-paretic difference with ankle subscore change | 78 | −0.171 (−0.393 to 0.054) |  |  | 0.135 | 0.203 |  |

Note. The four-item FMA-LE ankle subscore was calculated as the sum of four ankle-related FMA-LE items, maximum score 8. Change was defined as discharge minus baseline. Panel C reports Spearman correlations between ankle subscore change and sEMG changes; q values were computed across the three associations. FMA-LE, Fugl-Meyer Assessment of Lower Extremity; sEMG, surface electromyography; CI, confidence interval.
